# Inter-rater agreement in trait judgements from faces

**DOI:** 10.1371/journal.pone.0202655

**Published:** 2018-08-17

**Authors:** Robin S. S. Kramer, Mila Mileva, Kay L. Ritchie

**Affiliations:** 1 School of Psychology, University of Lincoln, Lincoln, United Kingdom; 2 Department of Psychology, University of York, York, United Kingdom; Bournemouth University, UNITED KINGDOM

## Abstract

Researchers have long been interested in how social evaluations are made based upon first impressions of faces. It is also important to consider the level of agreement we see in such evaluations across raters and what this may tell us. Typically, high levels of inter-rater agreement for facial judgements are reported, but the measures used may be misleading. At present, studies commonly report Cronbach’s *α* as a way to quantify agreement, although problematically, there are various issues with the use of this measure. Most importantly, because researchers treat raters as items, Cronbach’s *α* is inflated by larger sample sizes even when agreement between raters is fixed. Here, we considered several alternative measures and investigated whether these better discriminate between traits that were predicted to show low (parental resemblance), intermediate (attractiveness, dominance, trustworthiness), and high (age, gender) levels of agreement. Importantly, the level of inter-rater agreement has not previously been studied for many of these traits. In addition, we investigated whether familiar faces resulted in differing levels of agreement in comparison with unfamiliar faces. Our results suggest that alternative measures may prove more informative than Cronbach’s *α* when determining how well raters agree in their judgements. Further, we found no apparent influence of familiarity on levels of agreement. Finally, we show that, like attractiveness, both trustworthiness and dominance show significant levels of private taste (personal or idiosyncratic rater perceptions), although shared taste (perceptions shared with other raters) explains similar levels of variance in people’s perceptions. In conclusion, we recommend that researchers investigating social judgements of faces consider alternatives to Cronbach’s *α* but should also be prepared to examine both the potential value and origin of private taste as these might prove informative.

## Introduction

It has often been argued that we ‘automatically’ form first impressions from photos of faces. Evidence for this automaticity comes from studies which have shown that impressions can be reliably formed at very short exposure durations [1,2], and are formed incidentally without instruction [3]. Ratings on social dimensions such as trustworthiness, attractiveness, and competence have been shown to influence dating [4], political [5,6], economic [7,8], and even court decisions [9,10].

One interesting and commonly reported aspect of social evaluation is that people seem to agree with each other’s social trait ratings despite limited evidence supporting their accuracy ([11] cf. [12,13]). This high consensus implies that our judgements are based on certain types of physical information in the face, and all current social evaluation models use data-driven approaches to extract this information (e.g., deriving key social dimensions from the ratings themselves [14]). Most studies report a high Cronbach’s *α* to show high agreement between raters. This, however, is not necessarily the best way of measuring agreement, or even the correct use of Cronbach’s *α*. Here, we will first review evidence for inter-rater agreement in first impressions across age, race, and culture. We will then discuss the most widely-used measure of agreement in the social literature, Cronbach’s *α*, and finally focus on alternative measures of rater agreement with the potential to address additional aspects of rating agreement such as individual participant reliability.

### Inter-rater agreement

High inter-rater agreement in the attribution of social traits has been reported as early as the 1920s. In an attempt to refute the study of phrenology using statistical evidence, and thus discourage businesses from using it as a recruitment tool, Cleeton and Knight [15] had members of national sororities and fraternities rated for a number of social traits (e.g., leadership, frankness, intelligence, etc.) by both close associates and casual observers. Phrenology-based measurements of the ratees’ heads such as head width, forehead breadth, and distance between the eyes, were not related to differences in social ratings, although there was very high agreement between ratings of both familiar and unfamiliar observers. Such findings imply that while there certainly is some information in the face people use when making social judgements, this information is not captured by simple distances between the features of the face.

High inter-rater agreement has been reported consistently for a number of social traits ([16,17] see also [18] for a meta-analysis on attractiveness), as well as across different demographic groups. Cogsdill and colleagues [19,20], for example, showed significant consensus between children aged 3–10 and adult perceivers for ratings of trustworthiness, dominance, and competence. This was the case for ratings of both adults’ and children’s faces [20]. High inter-rater agreement has also been reported across different races and cultural groups (White, Black, and Asian raters [21]; White American and Chinese raters [22]; White American and Korean raters [23]). Here, not only did members of the same race agree on judgements of other race faces, but members of different races also gave similar ratings to the same faces. This high consensus was seen in both social attributes such as warmth, attractiveness, intelligence, and dominance, and traits more closely related to face shape such as age and babyfacedness.

Further evidence for cross-cultural inter-rater agreement on social dimensions comes from Zebrowitz and colleagues [24], who compared ratings that American and Tsimane participants attributed to each other. Despite the fact that the Tsimane are an isolated ethnic and linguistic group in the Bolivian Amazon, the authors reported relatively high within and between culture consensus for ratings of warmth, attractiveness, and dominance. Moreover, cross-cultural ratings have been shown to lead to similar social outcomes. Rule and colleagues [25], for example, report that evaluations of American political candidates by Japanese participants could predict the outcome of American elections and vice versa. The social trait associated with election success, however, differed across cultures–while American election outcomes were best predicted by ratings of power, a warmth dimension predicted the Japanese election outcomes.

Nevertheless, it should be noted that high levels of agreement do not mean complete agreement. Emotional expressions, and smiles in particular, have been closely related to ratings of warmth and trustworthiness [26], yet there is evidence that smiling is interpreted somewhat differently across cultures [27,28]. Studies have also shown own-race favouritism in first impressions [29] and lower consistency between male and female perceivers [30].

### Cronbach’s α and private vs shared taste

Despite the vast evidence that people form similar first impressions from unfamiliar faces, there is some debate over the way to best measure this agreement, with more and more studies questioning the use of the most popular measure of agreement in the literature–Cronbach’s *α* [31]. Cronbach’s *α* was originally developed as a measure of reliability in psychometric tests but it is also the most widely used measure of scales’ internal consistency and, recently, of inter-rater reliability in the social evaluation literature [16,17,21,25]. Since its publication in 1951, Cronbach’s paper has been cited over 35,000 times, demonstrating its impact and widespread use. Despite its popularity, some researchers have expressed their concerns about the uses and misuses of Cronbach’s *α* both as a measure of internal consistency and inter-rater agreement [32–37]. They argue that *α* is representative of internal consistency under requirements which are very rarely met in psychological research and that it has been mistakenly interpreted as a measure of unidimensionality [38]. Importantly, while internal consistency is necessary for homogeneity, it is not sufficient. Moreover, what makes *α* problematic in social evaluation research is the fact that raters are treated as items, and so increasing the number of raters leads to an increase in the resulting agreement value. In the context of scale reliability, a test with more items is naturally likely to be more reliable, but there is no reason why using more raters in social evaluation experiments should lead to higher inter-rater agreement values.

Cronbach’s *α* captures the average social trait rating attributed to a certain face by the total population of perceivers. Thus, a high *α* value means only that the ratings given are able to estimate those of the general population. It does not follow, however, that the standards used by perceivers to make such judgements are mostly shared. Hönekopp [36] points out that such an approach ignores an important proportion of variance in social ratings, i.e., within-person variability or how much raters agree with themselves. He argues that it is only when we consider both within- and between-person variability that we can establish to what extent first impression rating standards are shared or private. Hönekopp’s beholder index (*bi*) measure for ratings of attractiveness showed that even when Cronbach’s *α* was very high (*α* = .95), there could be an equal contribution of shared and private variance in ratings [36]. Indeed, recent multilevel modelling techniques have also provided evidence that shared and private tastes contribute equally to judgements of attractiveness [39]. Such findings oppose the dismissal of private standards as noise and highlight the importance of exploring individual differences in social evaluation. Therefore, whether people agree on their first impressions and how much these impressions are based on private and shared standards are two separate questions that require different measures, and should both be addressed by the current social evaluation literature.

### Measures of agreement

We have introduced both Cronbach’s *α* and Hönekopp’s *bi* as measures used for quantifying inter-rater agreement. However, there are several additional candidates that have been reported previously by researchers, although far less frequently than Cronbach’s *α*. We therefore provide further details on all the measures we include in the current work.

#### Cronbach’s α

Most researchers typically report Cronbach’s *α* as a measure of reliability among raters. This value is equivalent to the intraclass correlation coefficient (ICC; for a useful guide, see [40]) for a two-way mixed effects model (i.e., both the identities and the raters are subsets of populations). In addition, it is the specific case where the consistency in ratings is considered (how the identities are ordered rather than the absolute agreement in ratings) and we want to know the reliability of the average rating [41]. One can also think of this measure as the mean of all split-half reliabilities, as long as the differences in item standard deviations are taken into account [31,32]. As noted above, the main criticism with this measure is that, for social evaluation research, simply increasing the number of raters produces an increase in the resulting agreement value [32].

#### Intraclass correlation coefficient—ICC(*A*,*k*)

It is important to consider the difference between consistency and absolute agreement. Raters may agree on the general order of faces with respect to a particular trait (face A is more attractive than face B) but they may also show systematic differences in terms of the absolute ratings they give (rater 1 gives higher values than rater 2 for both faces). Whether researchers are interested in these absolute differences or not, given that ratings are typically averaged across raters, will depend on the particular question under investigation. If the purpose is to rank faces using their average ratings and choose the highest and lowest subset, for example, then the difference among raters may have little effect as long as consistency is high. However, if the purpose is to select faces which are rated above or below a predefined absolute score, or to consider their absolute (mean) rating in some other way, then raters need to show absolute agreement for this to be meaningful. Readers should be aware that Cronbach’s *α* ignores any absolute differences and only considers consistency. For consideration, we report values of absolute agreement–ICC(*A*,*k*)–for comparison with Cronbach’s *α*, which is also termed ICC(*C*,*k*).

#### Average leave one out

Another method for quantifying inter-rater agreement is to correlate each participant’s ratings with the mean of the remaining participants [42–44]. If we do this for every participant and then average the correlations together, we produce a value where higher indicates greater agreement within the group. Intuitively, this method quantifies how much we can expect any individual to agree with the remaining sample of raters.

#### Coefficient of concordance–Kendall’s *W*

Kendall [45,46] developed a measure, termed *W*, which is proportional to the average rank-order correlation among all pairs of raters. This nonparametric statistic produces higher values for raters that demonstrate higher agreement, and has previously been used to show that likeness ratings demonstrate little agreement across raters [47].

#### Within-participant reliability–Item retest

One important issue to consider when measuring the agreement among raters is their agreement with themselves. If an individual rater is unreliable or inconsistent across different sessions, for example, then this places limitations on how much other raters can hope to agree with them. We are able to quantify within-person reliability here by asking each rater to provide two sets of ratings for the same images. By correlating each rater’s first and second responses, we can obtain a measure of test-retest reliability [48].

#### Average inter-rater agreement

If we were to consider only two raters, we could simply correlate the two sets of ratings using either the values or their rank orders and this would provide a measure of their agreement. Extending this idea to groups of raters, we can use modern computing power to allow us to calculate the correlation between every possible pair of raters–the average inter-rater agreement [36,42,49,50]. We can then compare inter-rater agreement with test-retest reliability (how much raters agree with themselves), which can be thought of as an upper bound on how much we can expect raters to agree with each other.

#### Beholder index—*bi*

Based on generalisability theory [51], Hönekopp [36] put forward a measure that aimed to quantify the amount of shared versus private taste observed within attractiveness ratings. His beholder index represents the proportion of meaningful variance stable across time (using two sets of ratings provided by each rater) that arises from private taste. Specifically, by estimating variance components, one can then interpret these as observed variance attributed to participants, stimuli, time, and their interactions. The repeated measure (rating the set of faces twice) is necessary in order to separate these components from the residual. Here, we calculated *bi* in line with Hönekopp’s [36] approach.

### The present work

Researchers often assume high rater agreement, with only a passing consideration as to the level of agreement across raters and whether it is meaningful [11]. Where agreement is quantified, a single measure of agreement is typically reported (most frequently Cronbach’s *α*), along with a statement that the observed amount justifies consideration of only the average rating for each face. As mentioned above, using the average rating in subsequent analyses can result in conclusions that may be misleading or even meaningless if individual differences are both large and potentially informative.

In the current work, we focus on issues that are rarely considered in depth in the social evaluation literature: 1) are people consistent in their trait ratings of faces? 2) do people agree with others on seemingly subjective impressions? 3) how best might we quantify inter-rater agreement in first impressions? and 4) what is the proportion of shared compared with private variance in ratings of socially-important traits other than attractiveness? We collected ratings on six traits (gender, age, attractiveness, trustworthiness, dominance, and parental resemblance) that were expected to vary significantly in their levels of inter-rater agreement.

Ratings of gender and age are both based on cues contained in the physical appearance of the face which leads to highly consistent as well as accurate judgements on those traits [52–56]. We therefore expect to find the highest inter-rater agreement and shared variance in ratings of gender and age in comparison with ratings on the other social dimensions.

For different social traits, there may be reason to predict different levels of shared compared with private variance in ratings. Dominance attribution has been closely associated with face structure, where faces with a higher width-to-height ratio are generally perceived as more dominant and aggressive [57,58]. Therefore, we might expect substantial shared variance and agreement for dominance ratings because, much like age and gender, the cues are in the physical structure of the face. However, research also points to significant individual differences in dominance judgements due to perceiver characteristics [59]. As such, we might predict a sizable influence of private taste also. Previous studies have shown that there is an evolutionary link between attractiveness and mate value [60–63], and so we would expect to observe more shared variance than private variance in attractiveness ratings, although perhaps less agreement in ratings than for dominance. Trustworthiness evaluation has been shown to rely, at least to an extent, on emotion overgeneralisation, where faces with even the most subtle resemblance to a happy expression are judged as more trustworthy than faces resembling an angry expression [26]. Trustworthiness judgements are also influenced by structural characteristics like width-to-height ratio [64]. Together, these stimulus qualities may lead to less variability in ratings and so more shared variance or agreement. However, individual differences also play a role, such as raters’ implicit race attitudes [65]. To sum, there is good reason to expect that dominance, attractiveness, and trustworthiness should all show substantial influences of both shared and private taste, although it is less clear as to the differences between these traits with respect to the two sources of variance.

Finally, parental resemblance was specifically selected to produce lower inter-rater agreement. Here, participants were asked to rate how much each image resembled their own parents. We expect much lower agreement compared to all other traits, as well as a higher contribution of private taste, because there is no reason for participants’ parents to each resemble the rated faces to the same extent.

In addition, we consider the potential influence of familiarity on levels of agreement for the first time. Much research has now established how important face familiarity is when performing recognition or matching tasks [66]. However, it remains untested as to whether familiarity affects the amount of agreement observed across raters. We hypothesise that previous familiarity with identities may result in lower levels of inter-rater agreement because, although responses are made to the same images, each rater’s personal perception could be influenced by their previous experience with the identity. In addition, this may be more evident for some evaluations than others–perhaps the perceived gender of a person, for example, will be stable no matter the amount of prior experience or familiarity gained. By presenting both familiar and unfamiliar identities in the current work, we consider this question.

## Method

### Participants

In order to include a participant’s data, we required that they recognise at least 50 familiar identities of the 100 viewed. For each of six traits that were rated (with each participant rating one trait only), we continued to collect data until a sample of 10 participants fulfilled this criterion. In total, this meant that 71 students participated. All were undergraduates at the University of York, and took part in exchange for a monetary payment or course credits. Of our final sample of 60 participants (53 women; age *M* = 19.1 years, *SD* = 0.86 years), 57 reported their ethnicity as White or White mixed.

Informed consent was obtained prior to participation. The University of York’s psychology department ethics committee approved the experiment, which was carried out in accordance with the provisions of the World Medical Association Declaration of Helsinki.

### Stimuli

Photographs were downloaded from Google’s image search. An initial set of 400 White identities (half women; one image of each) was collected. Two hundred were chosen to be familiar to participants and included international actors, singers, etc., while the other 200 included unfamiliar identities (national celebrities only famous within their own countries). After initial pilot testing with colleagues to confirm familiarity, we produced a final set of images comprising 50 familiar women, 50 familiar men, 50 unfamiliar women, and 50 unfamiliar men. Although age was not available for these identities at the time the images were taken, we estimate a range of approximately 20–70 years old.

**Fig 1:**
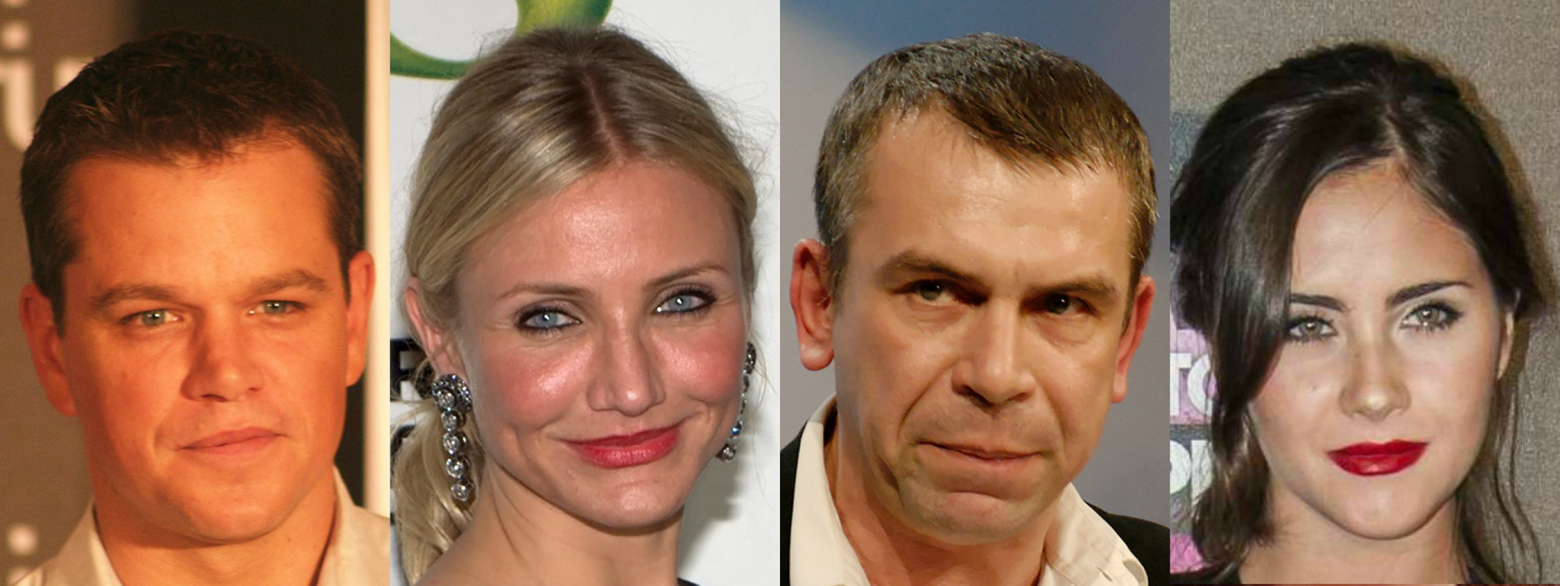
Example images. Images shown here are representative of those presented in the experiment, although copyright restrictions prevent publication of the original images used. Image attributions from left to right: Miguel Ángel Azúa García (Own work) [CC BY 3.0], David Shankbone (Own work) [CC BY 2.0], Marie-Lan Nguyen (Own work) [CC BY 2.5], Non Stop People España (Own work) [CC0 1.0].

All images were broadly front facing, upright, and displayed neutral or smiling expressions. In all other ways, they were unconstrained. Each image was cropped to include only the head and background (see Fig 1). Finally, images were resized to 380 x 570 pixels.

### Procedure

We used a fully crossed design, common to many studies in this field, where all images were rated by all raters for a given trait. Each participant was asked to rate all 200 images (half women, half familiar), presented in a random order, for one trait only. It was important that each participant only rated one trait in order that we would obtain independent measures of agreement for the six traits. In experimental designs where participants each rate images along several dimensions, there is the likelihood of contamination, whereby judging a face as attractive may influence subsequent judgement of that face on trustworthiness, for instance. Participants were assigned to traits based on the order in which they took part–collection on one trait continued until completion and then participants were assigned to the next.

The instruction presented onscreen throughout the experiment read “in this particular photograph, how X does the person look?” where X referred to the specific trait being rated. Five of the traits were gender (1 = very feminine, 7 = very masculine), age (1 = very young, 7 = very old), attractiveness (1 = very unattractive, 7 = very attractive), trustworthiness (1 = very untrustworthy, 7 = very trustworthy), and dominance (1 = very low, 7 = very high). The final trait was resemblance to a parent, so “in this particular photograph, how much does the person look like your mum/dad?” The instruction (“mum” or “dad”) was tailored to the particular sex of that image, and the rating scale was labelled ‘1 = not at all, 7 = very much’. In an attempt to avoid unwanted agreement simply due to age influences (i.e., all those with young parents would rate old faces as low resemblance), for images where the identity was older or younger than their parents, participants were instructed to imagine how much the person onscreen resembled their parent if that parent were a similar age.

These specific traits were chosen because we have reason to believe from prior literature that these traits should yield differing levels of agreement. Age and gender, where the cues are entirely in the facial structure, should yield the highest agreement, followed by dominance, attractiveness and trustworthiness, and finally parental resemblance, where there is no *a priori* reason for participants to agree. The prediction that these traits will yield differing levels of agreement gives us a testable prediction against which to compare the results of our different analysis measures.

After rating every image once, participants were instructed that there would be a second block of ratings, and could have a short break if they wished. Participants then carried out the same ratings task for a second time. That is, every image was rated on the same trait once more, with images presented in a new random order. Participants were not aware beforehand that they would be required to rate the images for a second time, in order to minimise the likelihood that they would try to remember and reproduce their first responses.

Upon completion of the two blocks of ratings, participants were shown every image once more (again, with the order newly randomised) and were asked to identify the person in the photograph. Specifically, we instructed participants to type in the person’s name or some identifying information that demonstrated recognition of the identity. We explained that simply typing “actor”, for example, would not be sufficient and that we required information particular to that person, e.g., the name of a film they starred in, the name of their spouse, etc. Participants were told that accuracy in spelling was not a concern. For those identities that were not recognised, participants were instructed to press the return key and continue to the next photograph. Both this task and the previous ratings tasks were self-paced.

## Results

Participants were unable to identify any of the unfamiliar people. However, for the familiar images, there were a number of identities that were not recognised. While each individual identified the majority of the 100 images, the specific images that were not recognised differed across participants. In order to carry out our ‘familiar’ analyses, we were only able to include images that all participants were familiar with. This was because many reliability measures require a fully crossed design (where all raters rate all images), and so we necessarily avoided missing values for particular images due to a lack of familiarity. The final number of images (i.e., those that all raters were familiar with) varied for each trait and is reported in S1 and S2 Tables.

**Fig 2:**
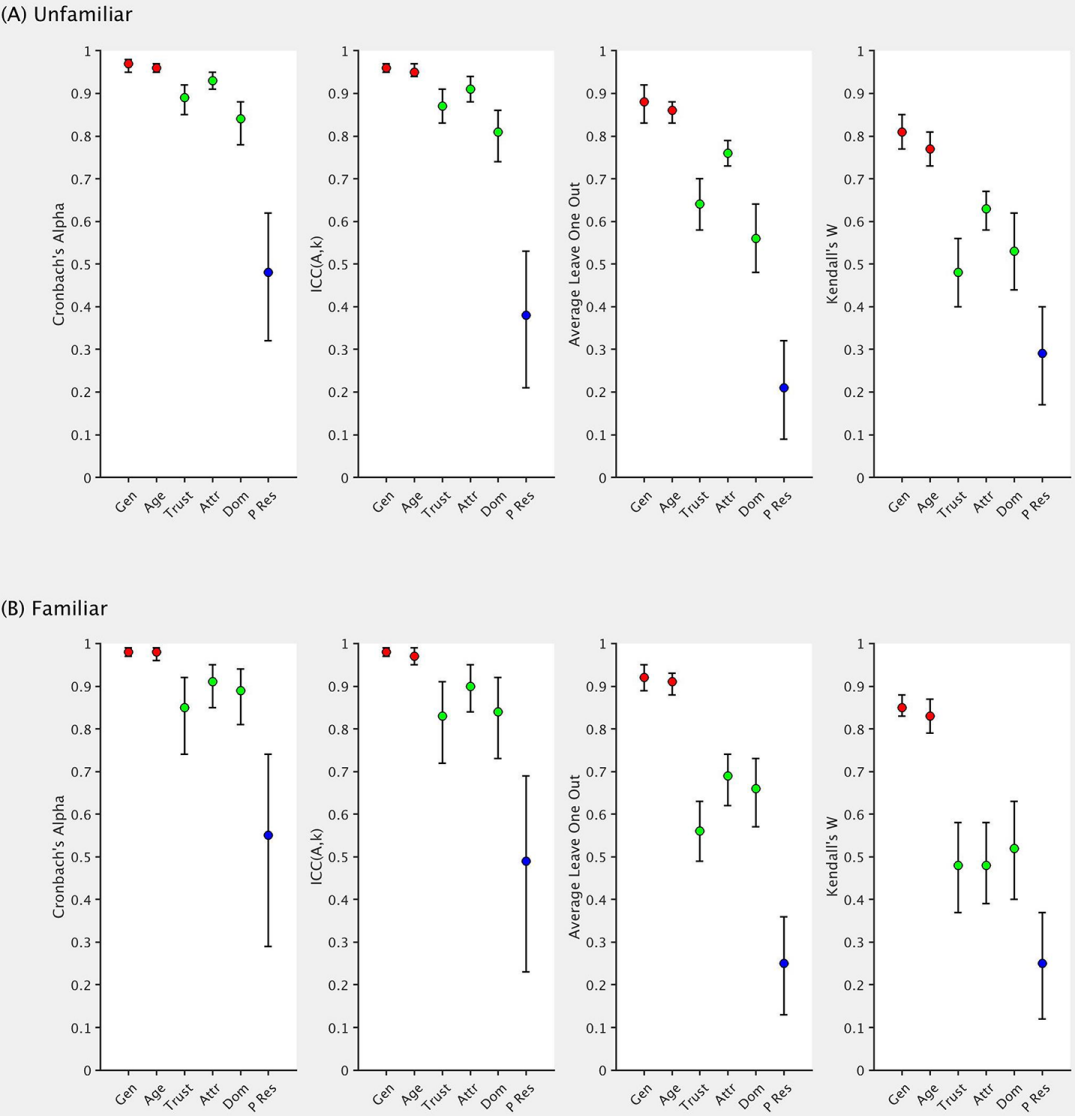
Different measures that are often reported in the context of scale or rater consistency. Error bars represent 95% confidence intervals. Traits are colour-coded to represent those we expected to show high (red), intermediate (green), and low (blue) inter-rater agreement. Gen = gender, Trust = trustworthiness, Attr = attractiveness, Dom = dominance, P Res = parental resemblance.

**Fig 3:**
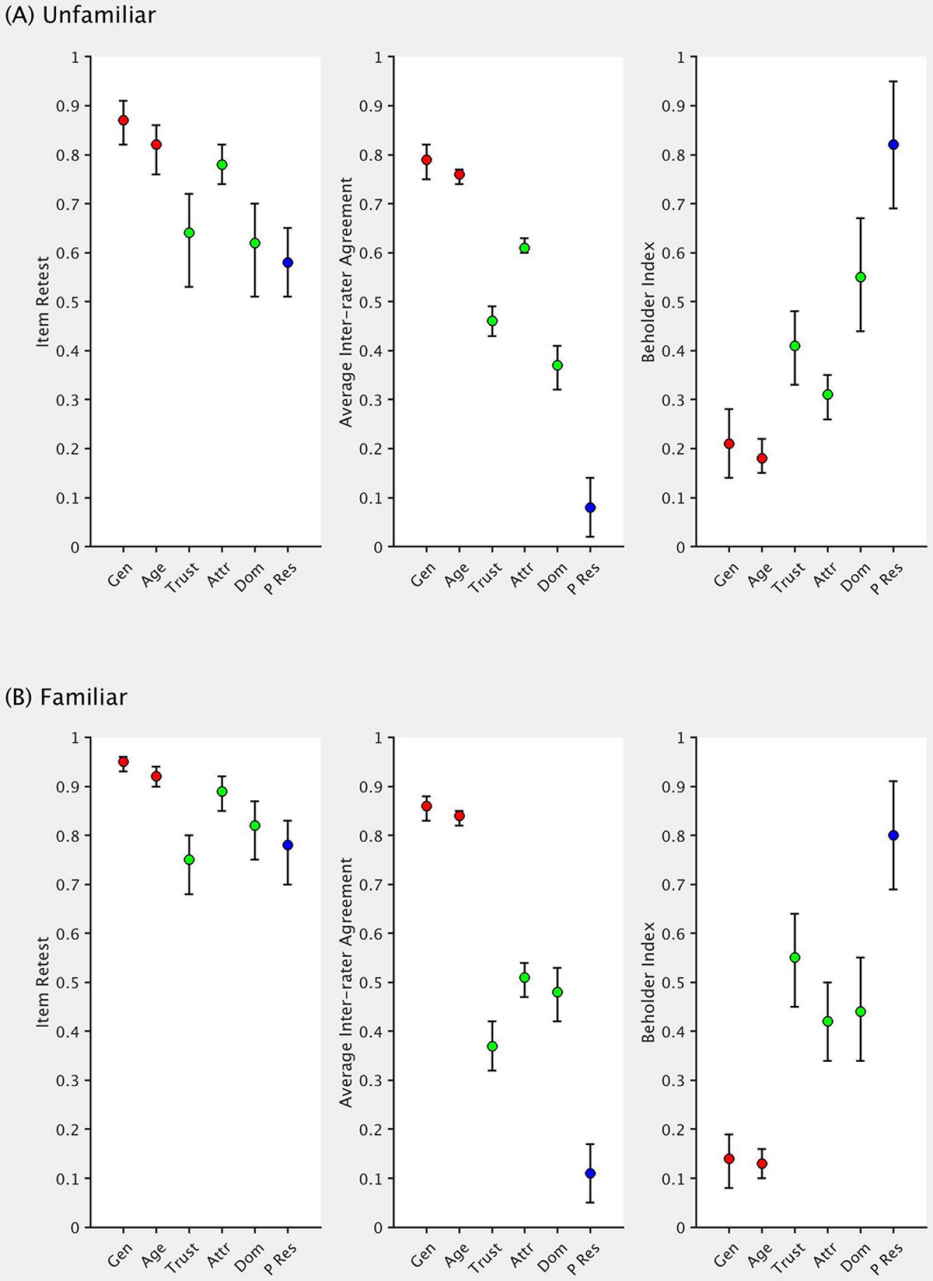
Within-participant reliability and estimates of shared and private taste. Error bars represent 95% confidence intervals. Traits are colour-coded to represent those we expected to show high (red), intermediate (green), and low (blue) inter-rater agreement. Gen = gender, Trust = trustworthiness, Attr = attractiveness, Dom = dominance, P Res = parental resemblance.

Below, we summarise our findings for seven different statistical measures. We present four of these in Fig 2 as an exploration of our first question described above–do raters agree in their evaluations of social traits? We then discuss three additional measures (Fig 3) that bear on our second question–how should we quantify shared agreement at the individual participant level and estimate the impact of differences in private taste? Finally, we consider whether familiarity showed any impact on our measures.

For many of the measures in Figs 2 and 3, articles typically report only point estimates. Here, we also provide confidence intervals in order to illustrate the precision of these measures, as well as allowing for statistical comparisons between traits. For both measures of intraclass correlation, IBM SPSS Statistics v25 provides values for the 95% confidence intervals. However, for the remaining measures, there is no established method for obtaining interval estimates. We therefore used a bootstrapping procedure in MATLAB, over 10,000 samples with replacement, in order to estimate standard errors, and subsequently, confidence intervals.

### Rater agreement

Fig 2 shows that the six traits appear differentiated with respect to Cronbach’s *α*. Age and gender clearly show high values, as one would expect. Interestingly, attractiveness is higher than both dominance and trustworthiness. Finally, and again as expected, parental resemblance shows the lowest reliability. It is worth noting that even this final trait shows a moderate level of reliability (0.48 for unfamiliar images) on this measure.

One of our focusses in the current work is whether the traits in our three tiers are well differentiated in line with predictions. Although age, which we expected to demonstrate high inter-rater agreement, falls very close to attractiveness (expected to show intermediate agreement) for unfamiliar faces, we find that statistically, these two values of Cronbach’s *α* significantly differ, *F*(89, 89) = 1.75, *p* = .005 [67].

We find the expectedly lower reliability of parental resemblance, in comparison with the other five traits, using only ten raters in the current experiment. However, as Hönekopp [36] noted, even traits with an inter-rater agreement of 0.10 (here, the average inter-rater agreement was 0.08 for unfamiliar images) would yield a Cronbach’s *α* of 0.90 if 80 raters were used. This is because simply increasing the number of raters will lead to an increase in the resulting reliability value [32]. It is important to note that Cronbach’s *α* was originally intended as a measure of test reliability, whereby lengthening a test produces a more reliable measure. When using Cronbach’s *α* in its current context as a measure of rater agreement, the number of test items is equivalent to the number of raters. This issue is one of the important criticisms of the use of this measure, where many researchers and readers may simply assume that a large value denotes high levels of agreement across raters. In contrast, no one would argue that an average inter-rater agreement of 0.10 demonstrated high agreement.

It is important to realise that Cronbach’s *α* has a limited definition. As already mentioned, this value refers to the reliability of the average rating and not single raters. In most cases, this is the appropriate measure, as researchers often average across raters to produce a value for each face [10,68–70]. However, interpretations are limited to the average rater and should only be generalised to other groups of raters and not single observers [40]. Simply, we may find that the average attractiveness rating is fairly reliable (here, 0.93 for unfamiliar images in Fig 2) but individual raters are less so (here, 0.78 when considering how well raters’ first and second ratings correlate).

Another consideration is the difference between consistency and absolute agreement, as mentioned in the introduction. We report values of absolute agreement—ICC(*A*,*k*)—in Fig 2 for comparison with Cronbach’s *α*. Here, we find that absolute agreement is typically similar but slightly lower than consistency.

We also investigated the average ‘leave one out’ correlation for each of the six traits. For each participant, we correlated their ratings with the mean of the remaining participants [42–44]. We then averaged these correlations together, producing a value where higher indicates greater agreement within the group. Average correlations were calculated by first performing Fisher’s *r* to *z* transformations, which correct for the skew in correlation distributions and provide an unbounded quantity that is approximately normal. The resulting *z* values were then averaged, and we finally applied *z* to *r* transformations [71]. In Fig 2, we see the same pattern across the six traits as for the intraclass correlation measures, although our three trait tiers are more clearly differentiated using this measure. The lowest value is given for parental resemblance, and the highest for age and gender. The other three traits fall somewhere in between. We also see this same three-tier pattern for Kendall’s *W* [45,46].

### Retest reliability and private vs shared taste

To obtain a measure of test-retest reliability, we correlated each rater’s first and second responses. Average correlations were then calculated as above. As Fig 3 illustrates, these values were high for all traits, and more so for familiar images (see below).

With this upper bound on how much we can expect raters to agree with each other, we then calculated the correlation between every possible pair of raters and averaged these values to produce the average inter-rater agreement [36,42,49,50]. Fig 3 shows that for only age and gender, the former measure approached the upper limit represented by the latter. To illustrate, with unfamiliar faces rated on gender, participants were highly consistent with themselves in their ratings across the two blocks (0.87), and we see a level of agreement between participants that approached this level (0.79). Therefore, for these two traits, there is a strong indication of shared taste, with almost all judgement variance that is stable over time explained by a shared standard [36]. In contrast, we see that dominance ratings demonstrated notably lower levels of agreement between participants (0.37) in comparison with their within-person stability over time (0.62), indicating a much larger influence of private taste.

We also investigated Hönekopp’s [36] beholder index. We find values of *bi*_1_ (the version of this index where absolute rater-score differences are assumed to be meaningless) for attractiveness that, along with previous research [36,42,50], demonstrate the important contribution of private taste. We find even larger contributions of private taste for ratings of trustworthiness and dominance. As expected, parental resemblance showed the largest amount of private taste since each participant was required to base their judgements on their own parents’ faces. Only age and gender showed very little private taste in raters’ judgements Previous research has shown that age is a trait that people can estimate accurately from the face (for a review, see [55]), and this would result in high agreement across raters. For gender, we would expect inflated levels of inter-rater agreement simply because accurate sex classification [53] should result in men’s faces receiving higher ratings than women’s faces on average, since men typically appear more masculine than women.

Although our measure of *bi*_1_ for judgements of unfamiliar face attractiveness in the current work (0.31) is notably lower than Hönekopp’s [36] estimate (0.49 in Experiment 1), it is comparable with values produced in other work (0.41 in [42] and 0.36 in [50]). It may be that the stimulus set used here incorporated greater variability along certain dimensions (e.g., age, where Hönekopp’s [36] set was restricted to a range of 16–37 years old), which might explain the larger influence of shared taste. For example, our undergraduate student raters would typically agree in judging older faces as less attractive.

Both the average inter-rater agreement and the beholder index allow us to quantify the amount of variance in ratings due to shared taste for each of the six traits. However, the former is a correlation and requires the value to be squared, while the latter quantifies private taste and we must therefore calculate the complementary percentage. For this reason, we present these derived values in Table 1 for comparison.

**Table 1:** Estimates of shared taste.

	Derived from the average inter-rater agreement	Derived from *bi*_1_
**UNFAMILIAR FACES**		
Gender	62%	79%
Age	58%	82%
Trustworthiness	21%	59%
Attractiveness	37%	69%
Dominance	14%	45%
Parental Resemblance	1%	18%
**FAMILIAR FACES**		
Gender	74%	86%
Age	71%	87%
Trustworthiness	14%	45%
Attractiveness	26%	58%
Dominance	23%	56%
Parental Resemblance	1%	20%

*bi* = beholder index.

It is reassuring, as illustrated in Table 1, that the order of the six traits is almost identical for the two measures of shared taste. However, Hönekopp’s [36] beholder index provides substantially larger estimates in all cases, most likely due to the differing methods with which these measures are calculated (correlational analysis vs. variance components utilising repeated measures).

### Familiarity

As Figs 2 and 3 illustrate, familiarity appears to have little effect on the various measures of reliability/agreement in image ratings. In particular, the orders of the six traits remain virtually unaffected by this distinction. This result suggests, perhaps surprisingly, that even when raters base their judgements solely on the images (for unfamiliar identities, the picture provides the only information they can use), agreement is no higher than for familiar identities (where picture information may be combined with prior rater-specific experiences with that identity).

While the general pattern of results is similar for familiar and unfamiliar images, we find that raters show consistently higher retest reliability for familiar identities (see Fig 3). Participants may be basing their judgements on already-established opinions of the celebrities, making these readily accessible and more easily reactivated when carrying out the second set of ratings.

## Discussion

In the present work, we considered several methods of calculating inter-rater agreement, and quantified such measures for a variety of traits. We expected to see our six traits fall into three tiers regarding the amount of agreement–high (age, gender), intermediate (attractiveness, dominance, trustworthiness), and low (parental resemblance). For the most part, our measures supported this clear distinction, although not in all cases.

If we consider the results summarised in Figs 2 and 3, we see that Cronbach’s *α* (along with the ‘absolute’ form of the ICC) showed the least ability to discriminate between these three trait categories (with the exception of item retest). Although this measure is the most widely used in the face perception literature as a method for quantifying inter-rater agreement, we know that simply increasing the number of raters will lead to an increase in the resulting reliability value [32]. Here, only ten raters judged each trait (which is typically lower than most sample sizes for this type of task), and so with larger samples, we might predict that even less separation will be present as all values become inflated.

All four remaining measures of inter-rater agreement (average inter-rater agreement, beholder index, Kendall’s *W*, average leave one out) clearly differentiate between traits that we expected to show high, intermediate, and low levels of agreement. Indeed, the fact that all these measures identified this pattern provides further validation regarding our initial predictions when selecting these traits. Comparison across measures is difficult, and there remains no clear way to justify the use of one over the others. However, our results suggest that any one of these may prove suitable as an indicator of inter-rater agreement, while reporting all measures can only serve to inform readers and those carrying out future research.

Our results also shed some light on the amount of shared versus private taste for social perceptions in addition to (and in comparison with) attractiveness. While dominance and trustworthiness show similar levels of private taste with each other and with attractiveness (as suggested by the beholder index, for example), perhaps there is some suggestion that both traits may be a little more ‘private’ than attractiveness (see Table 1). There are no clear reasons why this may be the case, and further research is needed. However, if consistent and replicable differences were found, then this would certainly prove to be an interesting line of research.

That we see no clear effect of familiarity on measures of inter-rater agreement is perhaps surprising. On the one hand, more familiar faces may activate shared representations across raters, resulting in higher levels of agreement. On the other, each rater may have their own unique experiences with familiar faces, influencing their perceptions and causing lower agreement. To illustrate, my specific exposure to Liam Neeson (perhaps only his recent action roles) may result in my rating his image as more dominant in comparison with someone whose exposure is based solely on his earlier characters. Here, we found no clear pattern as a result of manipulating familiarity beyond higher retest reliability for familiar faces. This may be because 1) familiarity has little effect on inter-rater agreement; 2) familiarity affects different traits in different ways; or 3) participants were able to ignore their previous experiences with familiar faces and successfully limit their ratings to the specific photographs (as they were instructed to do in the current task). Perhaps asking participants to rate the person rather than the photograph may have led to stronger familiarity effects, and we suggest this as an interesting extension for the future.

In line with previous research, we found that attractiveness ratings were influenced by both shared and private taste, although perhaps less so by the latter [36,50]. This pattern also appears to be true for dominance and trustworthiness judgements. Given that private taste explains a significant proportion of the variance for both these traits, we recommend that researchers consider the mechanisms underlying these individual differences in perceptions. For example, evidence suggests that ratings of others’ dominance may be affected by the perceivers’ height and other measures representing their own dominance [59,72,73].

In the current work, we consider the agreement or consistency in ratings across raters. We do not address the separate issue of the validity, or accuracy, in ratings–that is, how closely the ratings measure the actual trustworthiness, age, etc., of the people in the photographs. Previous research has shown that unfamiliar observers are able to accurately estimate age from face photographs [55], and there is also evidence demonstrating accuracy (although typically at low levels) for more complex social traits like trustworthiness [64,74] and dominance [75,76]. Of course, the nature of the particular photograph chosen for each identity plays a large role in subsequent judgements [77,78], and so studies addressing the accuracy of such judgements must necessarily take this into consideration.

In conclusion, our results highlight some of the issues associated with assuming/reporting high inter-rater agreement based solely on the use of Cronbach’s *α* as a metric. Further, several alternatives are described, all of which appear to provide better measures of inter-rater agreement in that they more successfully discriminated between traits that should *a priori* demonstrate different levels of agreement. In addition, we show that familiarity does not appear to influence inter-rater agreement. Finally, we provide the first investigation of the importance of shared versus private taste with regard to the fundamental social evaluation dimensions, trustworthiness and dominance, with our results suggesting levels comparable with attractiveness, and marginally more influence of shared taste (as measured with *bi*) in all three traits. Given the increasing popularity of research involving face perception and social traits, we strongly recommend the use of alternative/additional measures of inter-rater agreement, while being open to the idea that raters may not show high levels of agreement and that this might prove informative of itself.

## Supporting information

S1 Table Different measures that are often reported in the context of scale or rater consistency. (DOCX)

S2 Table Within-participant reliability and estimates of shared and private taste. (DOCX)

S1 Dataset Participants’ ratings for all traits. (XLSX)
