# Supplementary material for: Inter-rater agreement in trait judgements from faces
Source: PLoS One. 2018 Aug 17;13(8):e0202655. doi: 10.1371/journal.pone.0202655 (PMC6097668; doi:10.1371/journal.pone.0202655)
Supplement: S1 Table — (DOCX) [file pone.0202655.s001.docx]

# **Supplementary Table**

**S1 Table. Different measures that are often reported in the context of scale or rater consistency.**

|  | **ICC(*C,k*)**  **Cronbach’s *α*** | **ICC(*A,k*)** | **Average Leave One Out, *r*** | **Kendall’s *W*** |
| --- | --- | --- | --- | --- |
| **UNFAMILIAR FACES** |  |  |  |  |
| Gender (100) | 0.97 [0.95, 0.98] | 0.96 [0.95, 0.97] | 0.88 [0.83, 0.92] | 0.81 [0.77, 0.85] |
| Age (100) | 0.96 [0.95, 0.97] | 0.95 [0.94, 0.97] | 0.86 [0.83, 0.88] | 0.77 [0.73, 0.81] |
| Trustworthiness (100) | 0.89 [0.85, 0.92] | 0.87 [0.83, 0.91] | 0.64 [0.58, 0.70] | 0.48 [0.40, 0.56] |
| Attractiveness (100) | 0.93 [0.91, 0.95] | 0.91 [0.88, 0.94] | 0.76 [0.73, 0.79] | 0.63 [0.58, 0.67] |
| Dominance (100) | 0.84 [0.78, 0.88] | 0.81 [0.74, 0.86] | 0.56 [0.48, 0.64] | 0.53 [0.44, 0.62] |
| Parental Resemblance (100) | 0.48 [0.32, 0.62] | 0.38 [0.21, 0.53] | 0.21 [0.09, 0.32] | 0.29 [0.17, 0.40] |
|  |  |  |  |  |
| **FAMILIAR FACES** |  |  |  |  |
| Gender (30) | 0.98 [0.97, 0.99] | 0.98 [0.97, 0.99] | 0.92 [0.89, 0.95] | 0.85 [0.83, 0.88] |
| Age (22) | 0.98 [0.96, 0.99] | 0.97 [0.95, 0.99] | 0.91 [0.88, 0.93] | 0.83 [0.79, 0.87] |
| Trustworthiness (27) | 0.85 [0.74, 0.92] | 0.83 [0.72, 0.91] | 0.56 [0.49, 0.63] | 0.48 [0.37, 0.58] |
| Attractiveness (30) | 0.91 [0.85, 0.95] | 0.90 [0.84, 0.95] | 0.69 [0.62, 0.74] | 0.48 [0.39, 0.58] |
| Dominance (29) | 0.89 [0.81, 0.94] | 0.84 [0.73, 0.92] | 0.66 [0.57, 0.73] | 0.52 [0.40, 0.63] |
| Parental Resemblance (35) | 0.55 [0.29, 0.74] | 0.49 [0.23, 0.69] | 0.25 [0.13, 0.36] | 0.25 [0.12, 0.37] |

ICC = intraclass correlation coefficient. 95% confidence intervals are presented in square brackets. The final number of images included in the analyses are presented in brackets by the trait names.
