# Supplementary material for: Inter-rater agreement in trait judgements from faces
Source: PLoS One. 2018 Aug 17;13(8):e0202655. doi: 10.1371/journal.pone.0202655 (PMC6097668; doi:10.1371/journal.pone.0202655)
Supplement: S2 Table — (DOCX) [file pone.0202655.s002.docx]

# **Supplementary Table**

**S2 Table. Within-participant reliability and estimates of shared and private taste.**

|  | **Average within-participant reliability, *r* (Item Retest)** | **Estimated shared taste, *r***  **(Average Inter-rater Agreement)** | **Estimated private** **taste (*bi*_1_)** |
| --- | --- | --- | --- |
| **UNFAMILIAR FACES** |  |  |  |
| Gender (100) | 0.87 [0.82, 0.91] | 0.79 [0.75, 0.82] | 0.21 [0.14, 0.28] |
| Age (100) | 0.82 [0.76, 0.86] | 0.76 [0.74, 0.77] | 0.18 [0.15, 0.22] |
| Trustworthiness (100) | 0.64 [0.53, 0.72] | 0.46 [0.43, 0.49] | 0.41 [0.33, 0.48] |
| Attractiveness (100) | 0.78 [0.74, 0.82] | 0.61 [0.60, 0.63] | 0.31 [0.26, 0.35] |
| Dominance (100) | 0.62 [0.51, 0.70] | 0.37 [0.32, 0.41] | 0.55 [0.44, 0.67] |
| Parental Resemblance (100) | 0.58 [0.51, 0.65] | 0.08 [0.02, 0.14] | 0.82 [0.69, 0.95] |
|  |  |  |  |
| **FAMILIAR FACES** |  |  |  |
| Gender (30) | 0.95 [0.93, 0.96] | 0.86 [0.83, 0.88] | 0.14 [0.08, 0.19] |
| Age (22) | 0.92 [0.90, 0.94] | 0.84 [0.82, 0.85] | 0.13 [0.10, 0.16] |
| Trustworthiness (27) | 0.75 [0.68, 0.80] | 0.37 [0.32, 0.42] | 0.55 [0.45, 0.64] |
| Attractiveness (30) | 0.89 [0.85, 0.92] | 0.51 [0.47, 0.54] | 0.42 [0.34, 0.50] |
| Dominance (29) | 0.82 [0.75, 0.87] | 0.48 [0.42, 0.53] | 0.44 [0.34, 0.55] |
| Parental Resemblance (35) | 0.78 [0.70, 0.83] | 0.11 [0.05, 0.17] | 0.80 [0.69, 0.91] |

*bi* = beholder index. 95% confidence intervals are presented in square brackets. The final number of images included in the analyses are presented in brackets by the trait names.
